# Pan-cancer analysis reveals SGO1 as a potential cancer prognostic and immunological biomarker

**DOI:** 10.7150/jca.115334

**Published:** 2025-07-24

**Authors:** Yongqiang Wang, Xianming Long, Long Zhang, Fangfang Zhou, Miaochun Zhong

**Affiliations:** 1 Institutes of Biology and Medical Science, Soochow University, Suzhou, China.; 2 Department of Rheumatology and Immunology, The First Affiliated Hospital of Soochow University, Suzhou, China.; 3 General Surgery, Cancer Center, Department of Breast Surgery, Zhejiang Provincial People's Hospital (Affiliated People's Hospital), Hangzhou Medical College Hangzhou, China.; 4 MOE Laboratory of Biosystems Homeostasis and Protection and Innovation Center for Cell Signaling Network, Life Sciences Institute, Zhejiang University, Hangzhou, China.

**Keywords:** SGO1, pan-cancer, tumor, prognosis, biomarker

## Abstract

Shugoshin 1 (SGO1) is primarily known for its critical functions in chromosome segregation during cell division, protecting cohesin complexes and ensuring accurate mitotic processes. Previous studies have reported SGO1's regulatory roles in isolated cancer types, but its pan-cancer significance and underlying mechanisms remain undefined. This study systematically investigates SGO1 in 33 cancer types, integrating multi-omics analyses and functional validation to reveal its role as a pan-cancer biomarker and therapeutic target. Using TCGA, GEPIA2, and HPA databases, we found SGO1 was significantly overexpressed in 19 cancer types compared to normal tissues. High SGO1 expression correlated with poorer overall survival (OS) and disease-free survival (DFS) in more than 10 cancers, validated by Kaplan-Meier analysis. Genomic analysis revealed frequent SGO1 mutations and DNA methylation dysregulation, while immune profiling showed associations with immune cell infiltration (B cells, CD8+ T cells) and PD-1/PD-L1 checkpoint genes. Protein-protein interaction and enrichment analyses uncovered BUB1 as a key co-expressed gene, suggesting a role in spindle checkpoint regulation. Functional assays in breast cancer cell line MDA-MB-231 and lung cancer cell line A549 showed SGO1 knockdown inhibited proliferation, migration, and invasion, with xenograft models confirming reduced tumor growth. Our findings establish SGO1 as a novel pan-cancer biomarker, linking its expression to tumor progression, immune evasion, and genomic instability. This study bridges bioinformatics with functional validation, offering new mechanistic insights and therapeutic avenues for SGO1-driven cancers.

## Introduction

Cancer is expected to become the leading cause of death and the single most important obstacle to improving life expectancy in countries around the world in the 21st century 1. Although the mortality rate of cancer patients in developed countries has slowed down in recent years, the mortality rate of cancer patients worldwide is still showing a significant increasing trend due to the limitation of the spread of prevention and control ideas and medical technology innovation 2. Accelerating the mechanism research of tumor development is very important to improve the cure rate of tumor. Currently, pan-cancer research has been widely applied to identify tumor molecular markers and signaling pathways. When combined with various omics analyses, it holds the promise of providing a more comprehensive and in-depth understanding of the molecular mechanisms underlying tumorigenesis and progression 3-5. Among various regulatory proteins involved in cell division and genomic stability, Shugoshin 1 (SGO1) has emerged as a pivotal player. The physiological role of SGO1 is to ensure chromosome stability by protecting the cohesion of sister chromatid centromeres, facilitating bi-directional attachment of kinetochores, and preserving centromeric cohesion during both mitosis and meiosis 6, 7. Recent studies have begun to uncover the broader implications of SGO1 beyond its traditional functions, suggesting its involvement in various cancer types. For example, SGO1 is down-regulated at both the transcriptional and protein levels in human colorectal cancer, and this down-regulation of SGO1 leads to the occurrence of chromosomal instability (CIN) 8. Notably, recent studies revealed SET overexpression drives centromeric cohesion defects by competitively displacing SGO1, independent of PP2A. Cohesion is rescued by SGO1 overexpression but not by SGO1-binding-deficient SET mutants, revealing the SET-SGO1 axis as a non-canonical CIN pathway in cancer 9. Other studies showed that increased SGO1 can promote the proliferation and invasion of prostate cancer through AKT pathway, highlighting the potential of SGO1 as a new therapeutic target for prostate cancer 10, 11. Meanwhile, the expression of SGO1 in lung cancer and triple-negative breast cancer (TBNC) has also been shown to be significantly correlated with tumor proliferation and metastasis 12-14. SGO1 can also be used as a drug target and prognostic indicator for sorafenib in the treatment of hepatocellular carcinoma (HCC) 15. Alterations in the expression and function of SGO1 have been linked to tumorigenesis, enhanced cell proliferation, and poor patient prognosis across multiple cancer types. Furthermore, SGO1 may contribute to the development of chemoresistance, complicating treatment regimens and posing significant challenges in cancer therapy. Despite these findings, the comprehensive role of SGO1 in pan-cancer remains inadequately characterized.

In this study, we conducted a systematic bioinformatics analysis using multiple databases to determine the biological function and prognostic significance of SGO1 in pan-cancer. We comprehensively explore the functional significance of altered SGO1 expression levels in pan-cancer from the perspectives of gene expression, prognosis value, genetic alterations, tumor immune micro-environment, genomic heterogeneity, SGO1-related genes and interaction protein networks, as well as enrichment analysis. Furthermore, we conducted *in vitro* and *in vivo* experiments in breast cancer cell line and lung cancer cell line to validate the association between tumor proliferation and metastasis and SGO1 expression. It was concluded that SGO1 was a reliable pan-cancer biomarker, and it had good diagnostic and prognostic value in a variety of cancers. The analysis and experimental results of this study provided an insight that could pave the way for novel therapeutic strategies targeting SGO1 in cancer treatment.

## Materials and Methods

### Analyses of gene expression

TIMER2.0 (http://timer.cistrome.org/) is a comprehensive resource for systematized analysis of immune infiltrates across diverse cancer types 16. We entered the SGO1 gene into the “Gene Expression Analysis” module of the TIMER2.0 website to examine the difference in the expression of SGO1 between the tumor and neighboring normal tissues in pan-cancer from The Cancer Genome Atlas (TCGA) database. For validation purposes, we used another genetic analysis database, the Gene Expression Profiling Interactive Analysis (GEPIA2) (http://gepia2.cancer-pku.cn/), containing samples from the TCGA and GTEx databases, for validation analysis 17.

### Immunohistochemistry staining

HPA (https://www.proteinatlas.org/) is a human proteome mapping database that contains information about human tissue and cell protein distribution. In order to analyze the difference in the expression level of SGO1 protein between tumor and normal tissues, we downloaded the immunohistochemical images of corresponding tumor tissues and their corresponding normal tissues from HPA 18.

### Analysis of subcellular localization

We used the HPA database to obtain immunofluorescence images of SGO1 subcellular localization.

### Survival prognosis analysis and relationship with clinical stage

The overall survival (OS) and disease-free survival (DFS) survival map data for SGO1 in various tumor types in TCGA database were obtained via GEPIA2 online website. The prognostic value of SGO1 mRNA and protein expression in pan-cancer was also assessed according to OS/relapse-free survival (RFS) using Kaplan-Meier plotter (http://kmplot.com/analysis/), an online database including gene expression data and clinical data. The GEPIA2 “Stage Plot” module was used to examine the relationship between SGO1 expression and pathological stages.

### Correlation of SGO1 expression with DNA methylation

UALCAN (http://ualcan.path.uab.edu/) is a comprehensive, interactive web portal that is used to conduct an in-depth analysis of TCGA gene expression data 19. In this study, UALCAN was used to investigate the promoter methylation level of SGO1 in pan-cancer.

### Genetic alteration analysis of SGO1

The cBioPortal web (https://www.cbioportal.org/) was used for genetic alteration information of SGO1. The type and frequency of SGO1 mutations in cancers were explored with the “cancer types summary and mutations” module. The mutations sites were obtained from “mutations” modules 20.

### SGO1 CNV profile in pan-cancer based on GSCA

Cancer gene set analysis (GSCA) platform (http://bioinfo.life.hust.edu.cn/web/GSCA/) is a TCGA database based integrated multiple omics data of the web server 21. In this study, the GSCA website was used to analyze the association of SGO1 CNV with mRNA expression in different tumors, and the association of SGO1 CNV with different tumor survival.

### Tumor microenvironment correlation analysis

To assess the immune environment, we used the TIMER2.0 database to analysis the infiltration immune cell scores of SGO1 associated with six major immune cells in pan-cancer. These include B cells, CD4+ T cells, CD8+ T cells, macrophages, neutrophils, and dendritic cells. The xCell method for immune infiltration cell analysis, immune checkpoint analysis, as well as the calculation of StromalScore, ImmuneScore, and ESTIMATEScore for each tumor type using the ESTIMATE algorithm, were all performed using the Sangerbox3.0 (http://sangerbox.com/) online platform 22.

### Genomic heterogeneity analysis

Tumor mutational burden (TMB), mutant-allele tumor heterogeneity (MATH), microsatellite instability (MSI), purity, homologous recombination deficiency (HRD), loss of heterozygosity (LOH), and ploidy are strongly associated with tumor gene mutations, and tumors with high TMB, MATH, MSI, purity, HDR, LOH, and ploidy generally have more gene mutations. The relationship between genomic heterogeneity and SGO1 gene expression was analyzed using Sangerbox3.0 platform.

### Gene set enrichment analysis

The STRING Database (https://string-db.org/) is an online resource focused on protein-protein interaction (PPI) data 23. It brings together information from different experiments and prediction methods to provide detailed data about the interactions between proteins. We used the STRING database tool to construct a network of the top ten interacting partners of SGO1. Subsequently, we performed KEGG and GO enrichment analyses on these proteins using the SRplot web platform (https://www.bioinformatics.com.cn/) 24.

### Cell culture, transfection, and reagents

HEK293T, MDA-MB-231, and A549 cells were originally from American Type Culture Collection (ATCC) and cultured in Dulbecco's modified Eagle's medium (DMEM) supplemented with 10% FBS (Cat No. FSP500, ExCell Bio). All cell lines were cultured in a humidifying atmosphere at 5% CO2 at 37 °C and are free of mycoplasma contamination. The shRNAs for SGO1 was constructed to the pLKO.1 plasmid. This plasmid was then co-transfected into HEK293T cells along with packaging plasmids. After 48 hours, the supernatant was collected and filtered through a 0.45 μm pore-size filter, then stored at -80°C for future use. The SGO1 shRNA had the following sequence: 5'-GAAGATCAGATACCTACTATT-3' and 5'- CCGCAAATTCCTCTTGAAGAA-3'.

### Quantitative real-time PCR (qRT-PCR)

Total RNA was prepared using the RNAiso Plus kit (Takara). A total of 1 μg of RNA was reverse-transcribed using the HiScript® II Q RT SuperMix ((R223-01, Vazyme Biotech co., ltd). Real-time PCR was conducted with ChamQ Universal SYBR qPCR Master Mix (Q711-02, Vazyme Biotech co., ltd). All target gene expression levels were normalized to *GAPDH*. The primers used for quantitative PCR were as follows: SGO1, forward: 5'-GGACCCCATCCACATCTTCG-3' and reverse: 5'-GGCCGTATGCAGTGAGTGAT-3'; GAPDH, forward: 5'-AGATCCCTCCAAAATCAAGTGG -3', reverse: 5'-GGCAGAGATGATGACCCTTTT-3'.

### Immunoblot analysis

Cells were lysed with 1 ml of lysis buffer (50 mM Tris-HCl pH 7.5, 150 mM NaCl, 2 mM EDTA, and 0.5% NP-40) containing protease inhibitors (Sigma) for 10 min at 4 °C. After centrifugation at 12 × 10^3^ × g for 15 min, the protein concentrations were measured, and equal amounts of lysate were mixed with sample buffer containing 1% SDS for 5 min at 95 °C. The antibodies used for immunoblotting (IB) were as follows: SGO1 (ab58023, Abcam, 1:1000 for IB), β-actin (AC038, ABclonal, 1:10000 for IB).

### Immunofluorescence and confocal microscopy

Cells were washed with PBS and fixed in 4% paraformaldehyde in PBS for 20 minutes. After permeabilization with 0.2% Triton X-100, cells were blocked with 3% BSA. Anti-SGO1 (ABclonal, A16174, 1:100 for IF), anti-β-Tubulin (ABclonal, AC021, 1:100 for IF) were used to stain the cells, followed by incubation with fluorescent-dye-conjugated secondary antibodies. DAPI (Sigma-Aldrich) was used to counterstain the nuclei. Images were acquired using a Zeiss LSM 880 confocal microscope system.

### Cell proliferation assay

MDA-MB-231 and A549 cells (1 × 10^5^) were inoculated on a 6-well plate and cultured for 3 days. The number of cells was counted at 24, 48 and 72 h, respectively, and the cell proliferation rate was calculated.

### Clone formation assay

MDA-MB-231 cells and A549 cells (600 cells) were placed in 6-well plates and cultured at 37 °C for 10 days until the lesions were obvious. The colonies were fixed with 4% formaldehyde and stained with 0.5% crystal violet. After staining, the colonies were washed with PBS and counted.

### Wound healing assay

Cells were seeded in 6-well plates and cultured until they reached approximately 90% confluence. A 10 μL sterile micropipette tip was used to gently make linear scratches on the surface of the adherent cells. The cells were then rinsed gently twice with phosphate-buffered saline (PBS) to remove detached cells. After further incubation for 24 hours, the healing of the cell scratches was observed and documented using an inverted microscope.

### Transwell migration and invasion assay

The 24-well transwell plates (8 μm aperture; Corning, USA) were pre-treated overnight at 4°C with or without 100 μL of Matrigel (Corning, USA). Cells were starved in FBS-free medium for 12 hours, then digested and centrifuged. The resulting cell pellet was resuspended in serum-free medium to a final concentration of 1×10^5^ cells/mL. A total of 200 μL of this cell suspension was seeded into the transwell chambers with or without Matrigel. In the lower chamber, 600 μL of medium containing 10% FBS was added. After 24 hours of incubation, cells were fixed at room temperature with 4% paraformaldehyde for 20 minutes and subsequently stained with 0.5% crystal violet for 10 minutes. Cell counts were then recorded.

### Subcutaneous xenograft experiment

Five-week-old BALB/c female nude mice were purchased from Beijing Vital River Laboratory Animal Technology Co., Ltd. The sh-SGO1 and sh-NC group MDA-MB-231 (5 × 10^6^) cells were inoculated subcutaneously into nude mice (n = 5 for each treatment group). Tumor size was measured every 4 days starting on day 7. After 31 days, the mice were euthanized and the tumor weight was measured. The animal experiment was approved by the Animal Experiment Ethics Committee of Soochow University.

### Statistical analysis

Data from biological triplicate experiments were presented with error bar as mean ± SD. Two-tailed unpaired Student's t-test was used for comparing two groups of data. Statistical significance was determined by p-values less than 0.05. The following annotations were used to illustrate significance: *p < 0.05, **p < 0.01, ***p < 0.001, and ****p < 0.0001.

## Results

### Analysis mRNA expression of SGO1 in human pan-cancer

**Figure 1:**
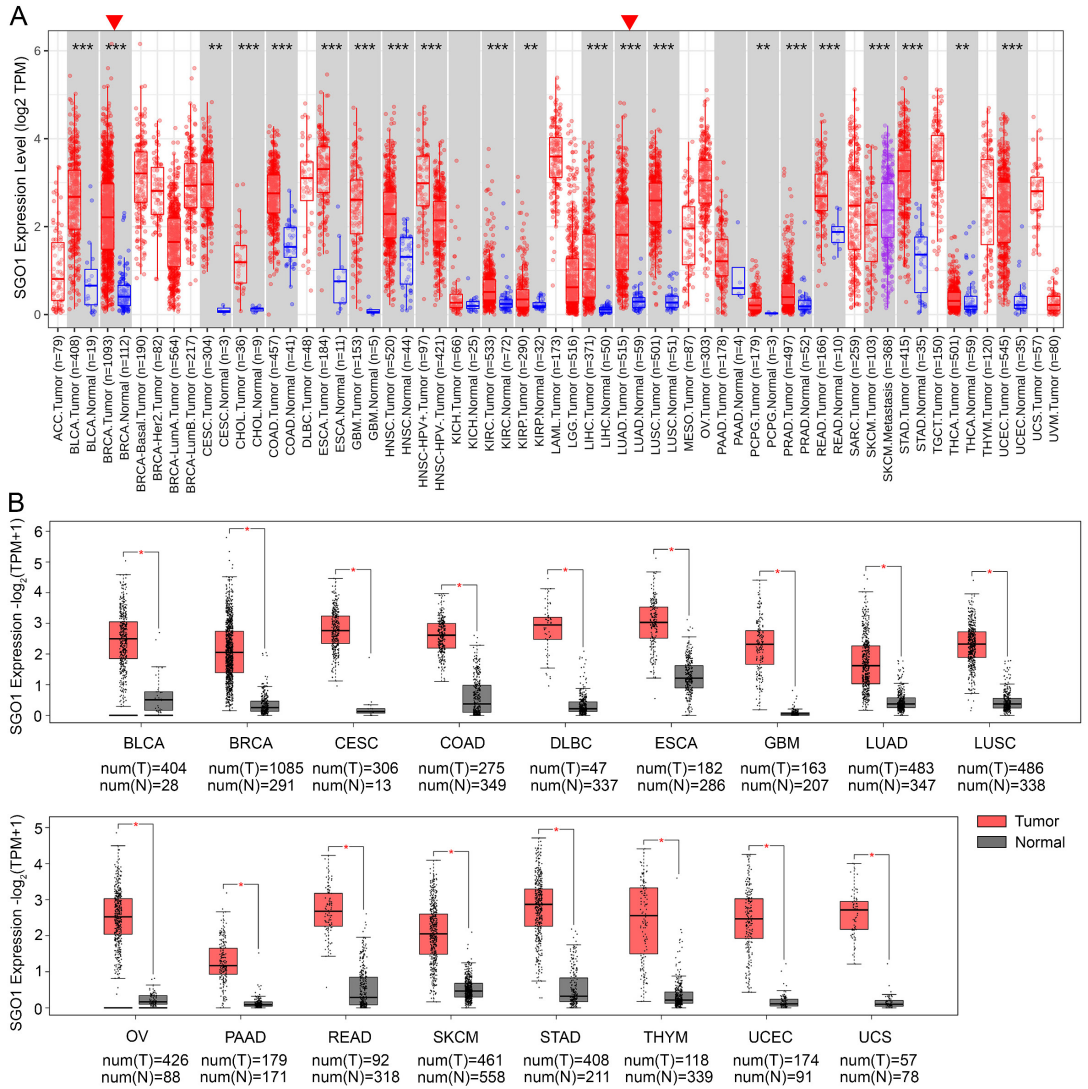
** Analysis mRNA expression of SGO1 in human pan-cancer. (A)** Analysis mRNA expression levels of SGO1 were analyzed in different cancer types from TCGA data in TIMER2.0. *p < 0.05, **p < 0.01, ***p < 0.001. **(B)** Differences in SGO1 expression between tumor and normal samples were analyzed using the GEPIA2. *p < 0.05.

The mRNA expression of SGO1 in human pan-cancer was analyzed based on the TCGA database by TIMER2.0 in log2 transformed TPM form. The results showed that SGO1 was highly expressed in BLCA (bladder urothelial carcinoma), BRCA (breast invasive carcinoma), CESC (cervical squamous cell carcinoma and endocervical adenocarcinoma), CHOL (cholangiocarcinoma), COAD (colon adenocarcinoma), ESCA (esophageal carcinoma), GBM (glioblastoma multiforme), HNSC (head and neck squamous cell carcinoma), KIRC (kidney renal clear cell carcinoma), KIRP (kidney renal papillary cell carcinoma), LIHC (liver hepatocellular carcinoma), LUAD (lung adenocarcinoma), LUSC (lung squamous cell), PCPG (pheochromocytoma and paraganglioma), PRAD (prostate adenocarcinoma), READ (rectum adenocarcinoma), STAD (stomach adenocarcinoma), THCA (thyroid carcinoma), and UCEC (uterine corpus endometrial carcinoma) compared with their adjacent normal tissues (**Figure 1A**). In addition, we also utilized the GEPIA2 database to analyze the expression of SGO1 across multiple cancer types, incorporating normal samples from the GTEx database to expand the sample size of normal tissues. Compared to normal tissues, the expression levels of SGO1 were significantly higher in 17 cancer types (**Figure 1B**). Based on the results presented above, we conclude that SGO1 is highly expressed in most of tumors.

### Expression and subcellular localization of SGO1 in cancers

The high expression of proteins plays a critical role in tumor development, encompassing various aspects such as cell proliferation, metabolic reprogramming, immune evasion, and drug resistance 25-27. We utilized the Human Protein Atlas (HPA) database to further investigate the expression differences of SGO1 between tumor and normal tissues by immunohistochemistry. The results showed that SGO1 expression was significantly elevated in most cancer tissues, including breast, lung, stomach, liver, colorectal, and ovarian cancers, compared with normal tissues (**Figure S1A**). Furthermore, we also found that SGO1 was localized in the nucleus of U-251MG, U2OS, and Rh-30 cells from immunofluorescence images in the HPA database (**Figure S1B**).

### SGO1 is associated with different stages of multiple tumors in pan-cancer

**Figure 2:**
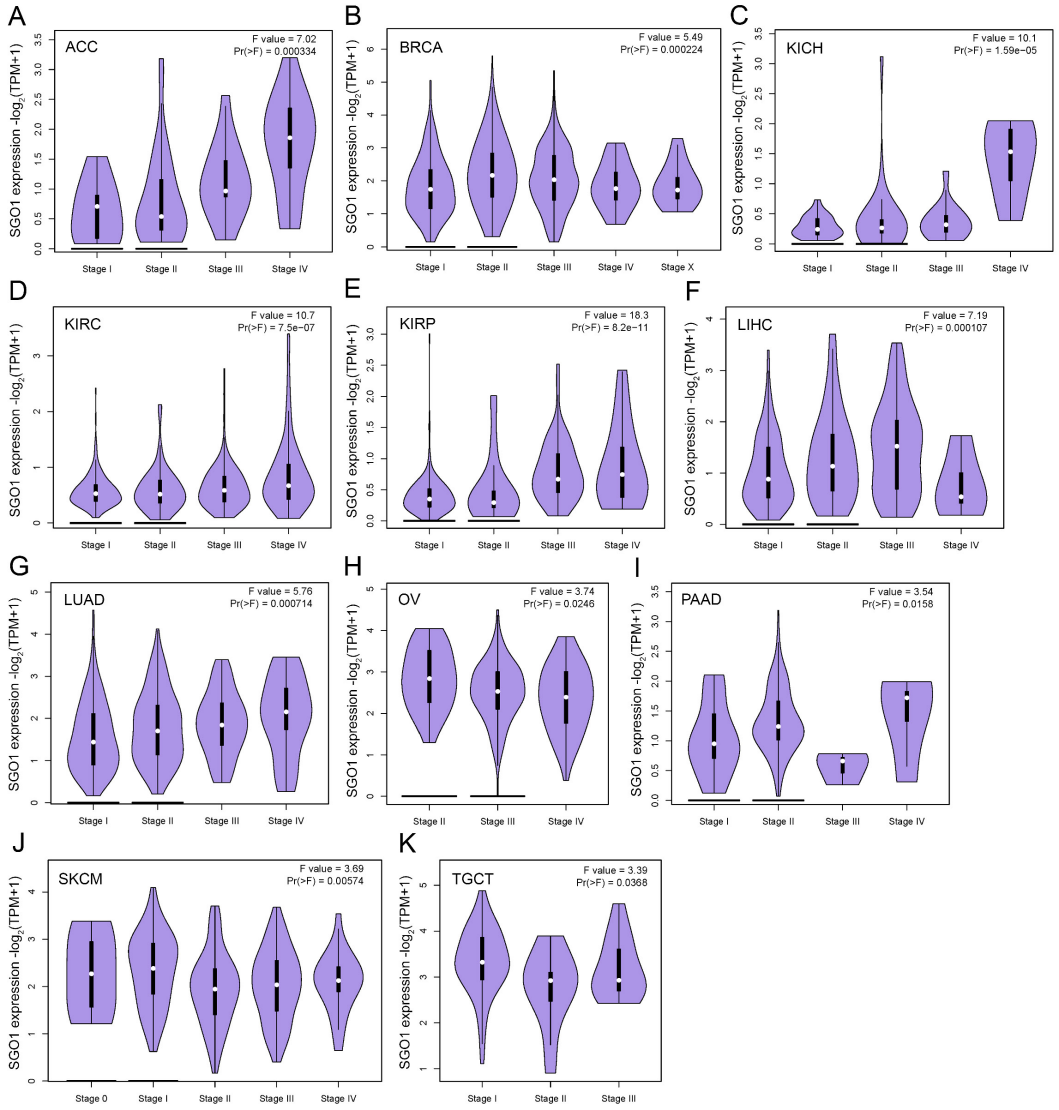
** SGO1 is associated with different stages of multiple tumors in pan-cancer. (A-K)** The correlation between SGO1 expression and the pathological stages of cancers, including ACC, BACR, KICH, KIRC, KIRP, LIHC, LUAD, OV, PAAD, SKCM, and TGCT using GEPIA2's “Stage Plot” module.

The high expression of specific proteins within tumors is often closely associated with the pathological stage of cancer. The correlation between the expression of SGO1 and the pathological stage of cancer was examined using GEPIA2's “stage Map” module. As shown in **Figure 2**, we found that SGO1 expression was correlated with pathological stages in several tumor types, including ACC (adrenocortical carcinoma), BRCA, KICH (kidney chromophobe), KIRC, KIRP, LIHC, LUAD, OV, PAAD (pancreatic adenocarcinoma), SKCM (skin cutaneous melanoma), and TGCT (testicular germ cell tumors).

### Prognostic value of SGO1expression in pan-cancer

**Figure 3:**
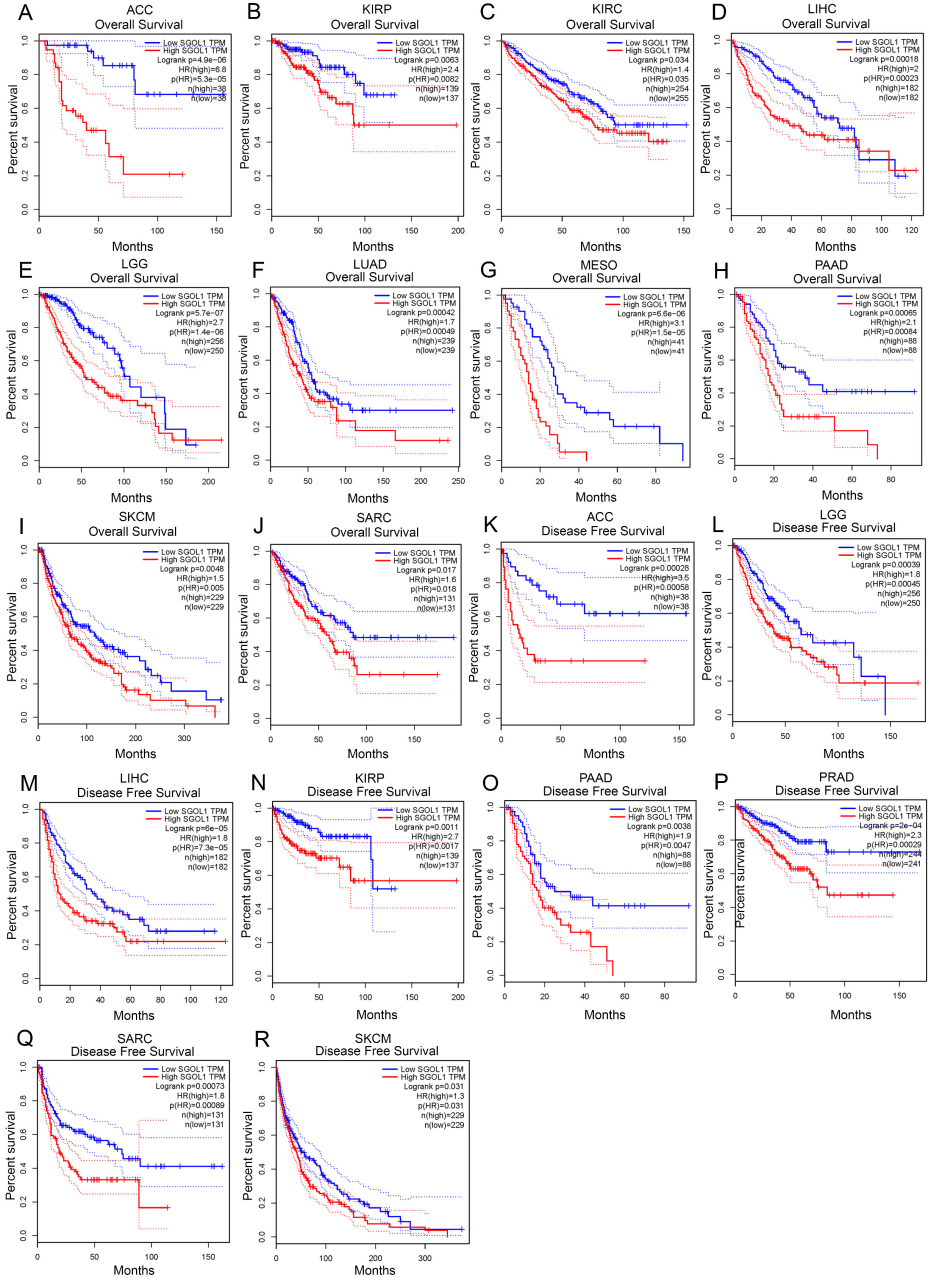
** Correlation between SGO1 expression and survival in different tumors. (A-J)** Analysis the correlation between SGO1 expression and overall survival (OS) in ACC, KIRP, KIRC, LIHC, LGG, LUAD, MESO, PAAD, SKCM, and SARC using GEPIA database. (K-R) Analysis the correlation between SGO1 expression and disease free survival (DFS) in ACC, LGG, LIHC, KIRP, PAAD, PRDA, SARC, and SKCM using GEPIA database.

Next, we investigated the prognostic value of SGO1 in pan-cancer. The GEPIA database was adopted to analyze the prognostic value of SGO1 in multiple cancers. We observed that high expression levels of SGO1 are significantly related poorer overall survival (OS) in most cancers, including ACC, KIRP, KIRC, LIHC, LGG (brain lower grade glioma), LUAD, MESO (mesothelioma), PAAD, SKCM, and SARC (sarcoma) (**Figure 3A-J**). Additionally, we also observed that a high expression level of SGO1 is markedly correlated with a poorer disease-free survival (DFS) in ACC, KIRP, LIHC, LGG, PAAD, PRAD, SARC, and SKCM (**Figure 3K-R**). In addition, we used the Kaplan-Meier Plotter database to examine the prognostic significance of SGO1 in pan-cancer. The results show that higher expressed SGO1 related to the poorer prognosis of UCEC (OS: HR = 1.83, P = 0.011), KIRC (OS: HR = 2.35, P = 2.2e-08), SARC (OS: HR = 2.07, P = 0.0022), KIRP (OS: HR = 5.68, P = 3.1e-10), BRCA (OS: HR = 1.57, P = 0.0065), LIHC (OS: HR = 2.32, P = 2.8e-06), PADC (pancreatic ductal adenocarcinoma) (OS: HR = 2.21, P = 0.00016), EAC (adenocarcinoma) (OS: HR = 2.49, P = 0.011), LUAD (OS: HR = 1.73, P = 0.00031) (**Figure S2A-I**). We also examined the association between higher SGO1 expression and recurrence free survival (RFS) of pan-cancer. SGO1 was observed to be significantly related to poorer RFS in KIRP (RFS: HR = 5.94, P = 9.5e-07), LICH (RFS: HR = 2.01, P = 2.5e-05), CESC (RFS: HR = 2.84, P = 0.029), EAC (RFS: HR = 2228392756.25, P = 0.0086), UCEC (RFS: HR = 1.79, P = 0.027), THCA (RFS: HR = 4.65, P = 0.00028), SARC (RFS: HR = 3.02, P = 0.00032), PADC (RFS: HR = 3.52, P = 0.0017), and LUAD (RFS: HR = 1.8, P = 0.035) (**Figure S3J-R**).

### DNA methylation level of SGO1 in pan-cancer

DNA methylation is associated with transcriptional inhibition, genomic imprinting, stem cell differentiation, embryonic development, and inflammation 28. Aberrant DNA methylation patterns are strongly associated with human diseases including cancer 29. Cancer cells are characterized by abnormal DNA methylation, including genomic hypomethylation and site-specific hypermethylation 30. By using UALCAN database, we examined the DNA methylation level of SGO1 in various tumors. The results showed that the methylation level of SGO1 in BLCA, LIHC, LUAD, LUSC, PRAD, TGCT, THCA, and UCEC tissues was significantly lower than that in normal tissues (**Figure S3A-H**). However, the methylation level of SGO1 was higher than that of normal tissues in CHOL, KIRC, and SARC (**Figure S3I-K**). Therefore, the expression level of SGO1 DNA methylation may be closely related to the pathogenesis of cancer.

### Mutation feature of SGO1 in pan-cancer

To elucidate the mutation characteristics and biological functions of SGO1 in tumor progression, the cBioPortal database was used to analyze genetic alterations of SGO1 in 10,967 tumor samples in pan-cancer. The results showed that SGO1 genetic variation was notably diverse and frequent, with alteration rates of 5.84%, 5.29%, 3.37%, 3.18%, and 2.54% in BLCA, UCEC, COAD, STAD, and KIRC respectively. Among them, the highest mutation rate (4.37%) was found in UCEC. Interestingly, in KIRC, the mutation type of SGO1 is “Deep Deletion” (**Figure S4A**). In various tumors, 106 variants of uncertain significance variants (VUS) in SGO1 were identified, including 76 missense mutations, 26 truncating mutations, and 2 spliced mutations. In addition, inframe mutations and fusion mutations were detected in only one case. We also found that missense mutation of SGO1 was the main type of genetic alteration, and M325Cfs*2/Nfs*9 alteration was detected in 3 cases of UCEC, 1 case of STAD, and 1 case of COAD, which could induce a frame shift mutation of the SGO1 gene (**Figure S4B**).

### The correlation analysis between SGO1 CNV with gene expression and survival

**Figure 4:**
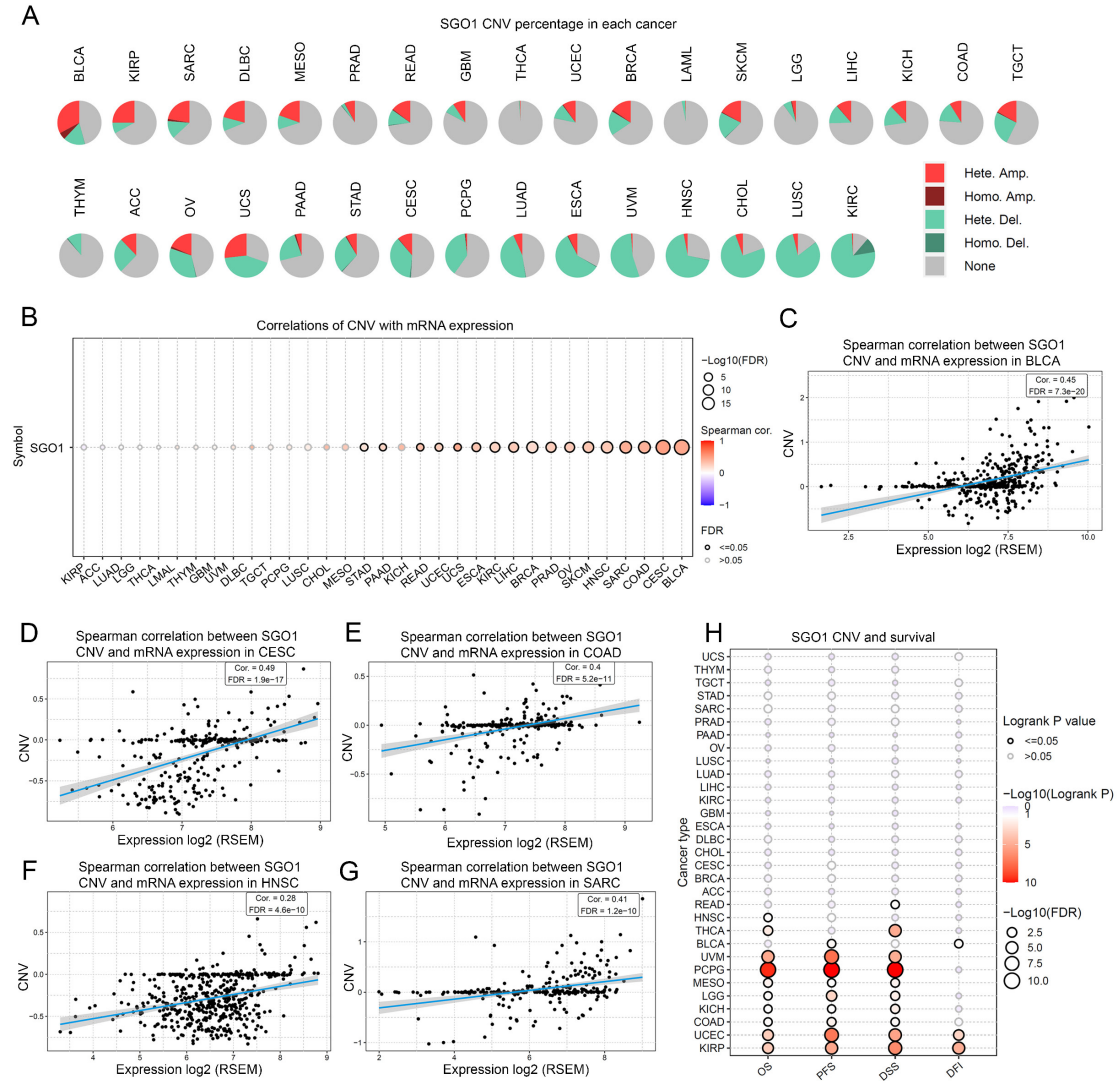
** The correlation analysis between SGO1 CNV with gene expression and survival. (A)** The CNV landscape of SGO1 in pan-cancer based on GSEA. **(B)** Analysis of connection between SGO1 CNV and mRNA expression. **(C-G)** The top five with the highest correlation scores between SGO1 CNV and mRNA. **(H)** Analysis the survival difference between SGO1 CNV and wide type in pan-cancer.

To examine the spearman association between SGO1 CNV and gene expression and survival, we performed an analysis of pan-cancer using the GSCA platform. According to the CNV pie chart, the main types of CNV in most tumors are heterozygous amplification and deletion (**Figure 4A**). There is a substantial positive connection between SGO1 CNV and mRNA expression in BLCA, CESC, COAD, SARC, HNSC, SKCM, OV (ovarian serous cystadenocarcinoma), PARD, BRCA, LIHC, KIRC, ESCA, UCS (uterine carcinosarcoma), UCEC, READ, PAAD, and STAD (**Figure 4B**). Based on the spearman correlation coefficient ranking, we present the results of the top five correlations (**Figure 4C-G**). Furthermore, we also found that a positively correlation between SGO1 CNV with disease free interval (DFI) (KIRP, UCEC, BLCA), DSS (KIRP, UCEC, COAD, KICH, LGG, MESO, PCPG, UVM (uveal melanoma), THCA, READ), PFS (KIRP, UCEC, COAD, KICH, LGG, MESO, PCPG, UVM, BLCA), and OS (KIRP, UCEC, COAD, KICH, LGG, MESO, PCPG, UVM, THCA, HNSC) (**Figure 4H**).

### Relationship of SGO1 expression and immunological environment in pan-cancer

**Figure 5:**
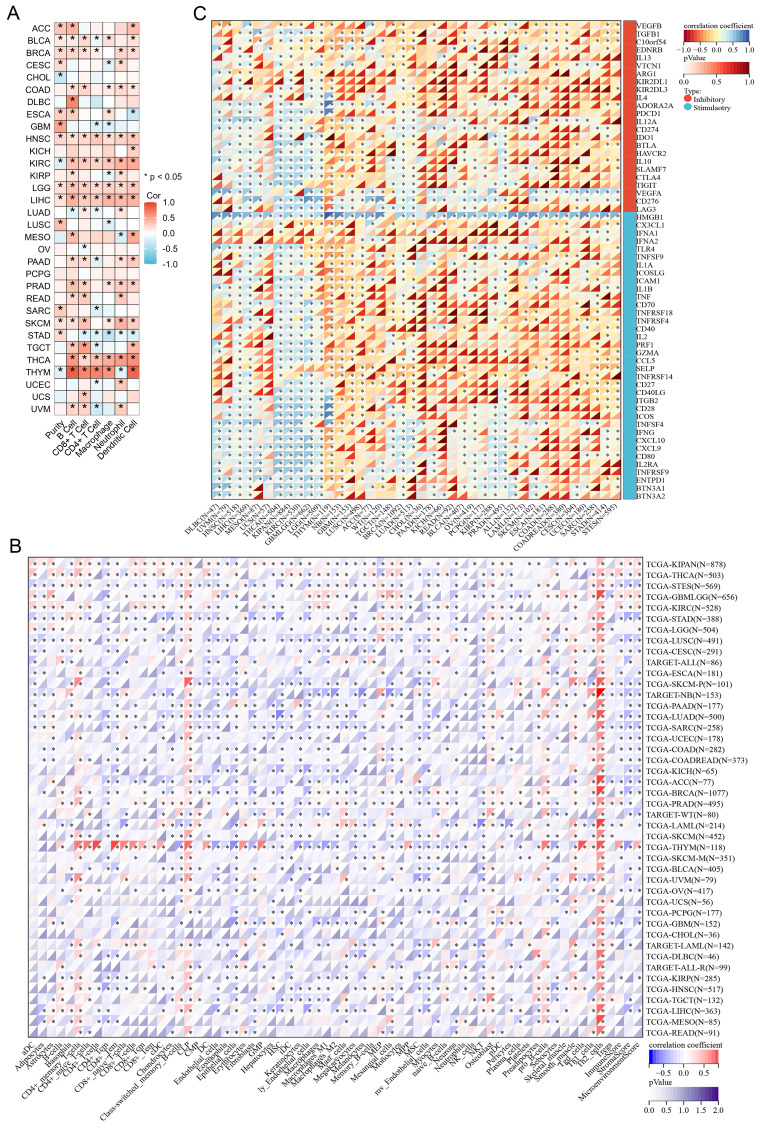
** Relationship of SGO1 expression and immunological environment in pan-cancer. (A, B)** Analysis the correlation between SGO1 expression and infiltrating immune cells in pan-cancer by TIMER method and xCell method. **(C)** Analysis the association of SGO1 expression and immune checkpoints in pan-cancer.

By using the TIMER2.0 tool, we found that the expression of SGO1 is significantly associated with the abundance of infiltrating immune cells in most cancers: 21 types of B cells, 19 types of CD8+ T cells, 16 types of CD4+ T cells, 16 types of macrophages, 19 types of neutrophils, and 18 types of dendritic cells (**Figure 5A**). We also observed that SGO1 expression affected tumor purity in 16 tumor types (**Figure 5A**). In addition, we employed the xCell method and found that the majority of the 38 immune cell subtypes were significantly associated with SGO1 expression across various tumor types. Among the different tumors, the correlation between SGO1 expression and Th2 cells was the strongest (**Figure 5B**). Immune checkpoint plays a critical role in tumor immune escape, innovative cancer therapy and prognosis prediction of patients with cancer. Next, we explored whether there was a relationship between SGO1 expression and the expression of these checkpoint genes based on the TCGA database. According to our results, SGO1 expression was highly correlated with the expression of immune checkpoint genes in most tumors, including transforming growth factor beta 1 (TGFB1), programmed cell death protein-1 (PDCD1, also known as PD-1), cytotoxic T-lymphocyte-associated protein 4 (CTLA4), and the CD274 (also known as PD-L1) genes (**Figure 5C**). Especially, the expression of SGO1 was positively linked with the majority of immune inhibitors, and immunostimulators in THCA, KIPAN (pan-kidney cohort), KIRC, and GBMLGG (glioma) (**Figure 5C**).

### Correlation analysis with the immune score using the ESTIMATE algorithm

**Figure 6:**
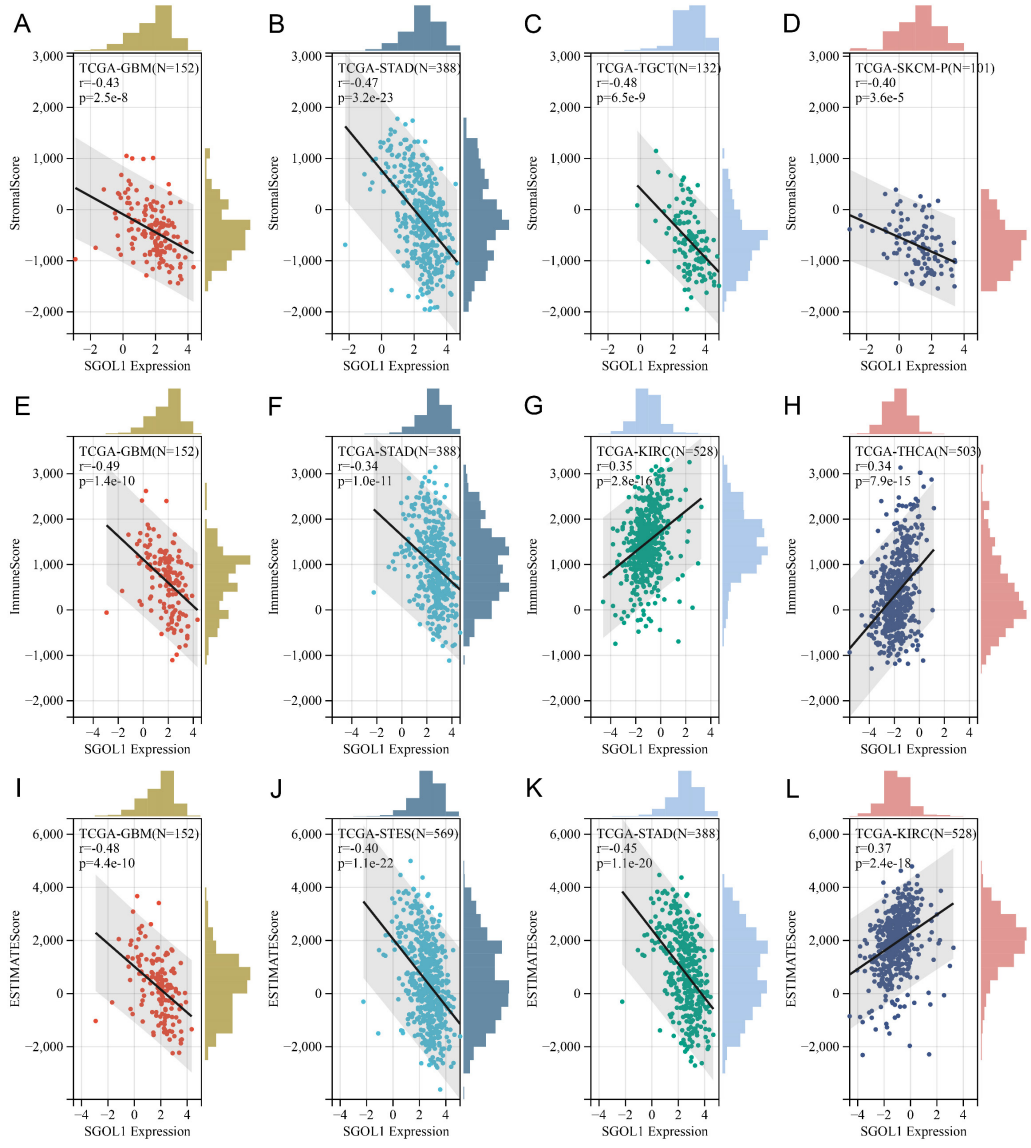
** Correlation analysis with the immune score using the ESTIMATE algorithm. (A-C)** Association of SGO1 expression and Stromal score, Immune score, and ESTIMATE score in pan-cancer.

Based on TGCA data set, we obtained immunoinfiltration scores (Stromal score, Immune score, and ESTIMATE score) for 9555 tumor samples from 39 tumor types. Spearman's correlation between genes and immunoinfiltration scores in each tumor was calculated using the Sangerbox online platform. We ranked all participating cancer types based on the absolute value of r from the aforementioned three score categories. The top four tumors most significantly correlated with expression of SGO1 were GBM, STAD, TGCT, and SKCM (Stromal score), GBM, STAD, KIRC and THCA (Immune score), GBM, STES (stomach and esophageal carcinoma), STAD, and SKCM (ESTIMATE score) respectively (**Figure 6A-L**). Therefore, the results indicated that SGO1 expression was tightly correlated with the extent of immune infiltration in cancers.

### Genomic heterogeneity analysis of SGO1

Tumor genomic heterogeneity is closely associated with the prognosis of cancer immunotherapy. Therefore, we investigated the relationship between SGO1 expression and four tumor heterogeneity indicators across pan-cancer, including tumor mutation burden (TMB), mutant allele tumor heterogeneity (MATH), microsatellite instability (MSI), purity, homologous recombination deficiency (HRD), loss of heterozygosity (LOH), and ploidy, to determine whether SGO1 can predict the response to immunotherapy. We found that SGO1 expression level was significantly positively correlated with TMB in GBM, GBMLGG, LGG, LUAD, LAML (acute myeloid leukemia), BRCA, STES, SARC, KIPAN, STAD, PRAD, HNSC, LUSC, READ, PAAD, SKCM, UCS, BLCA, ACC, and KICH, while negatively correlated in THYM (thymoma) (**Figure S5A**). Observing LUAD, BRCA, ESCA, STES, STAD, UCEC, LUSC, BLCA, we find SGO1 expression level positively tied to MATH but inversely related to GBMLGG, LGG, THYM, and LUSC (**Figure S5B**). In ESCA, STES, SARC, STAD, LUSC, LIHC, MESO, and BLCA, SGO1 expression level was positively correlated with MSI, whereas a negative correlation was observed in GBMLGG, KIPAN, and DLBC (lymphoid neoplasm diffuse large B-cell lymphoma) (**Figure S5C**). Notably, The SGO1 expression level was positively correlated with purity in GBM, GBMLGG, LGG, CESC, COAD, COADREAD (colon adenocarcinoma/rectum adenocarcinoma esophageal carcinoma), BRCA, ESCA, STES, SARC, STAD, PRAD, HNSC, LUSC, OV, SKCM, and ACC, while negatively correlated with purity in KIPAN, KIRC, and THYM (**Figure S5D**). In addition, we find SGO1 expression level was positively correlated with HRD in GBM, GBMLGG, LGG, LUAD, COADREDA, BRCA, STES, SARC, KIRP, KIPAN, STAD, PRAD, UCEC, KIRC, LUSC, LIHC, MESO, PAAD, SKCM, UVM, UCS, BLCA, ACC (**Figure S5E**). SGO1 expression positively correlates with LOH in GBMLGG, LGG, LUAD, BRCA, STES, SARC, KIRP, KIPAN, STAD, PRAD, UCEC, KIRC, LUSC, LIHC, MESO, PAAD, and BLCA, but negatively correlates with LOH in CESC, THYM, and TGCT (**Figure S5F**). Ploidy correlation analysis showed SGO1 expression positively correlates with LUAD, BRCA, STES, SARC, STAD, LUSC, THCA, TGCT, SKCM, and BLCA, but negatively correlates with GBMLGG, KIRP, and MESO (**Figure S5G**).

### Analysis of SGO1 in tumor stemness

Emerging evidence suggests that tumor stemness plays a crucial role in cancer progression and therapy resistance. To systematically investigate the association between SGO1 expression and pan-cancer stemness, we evaluated its correlation with four well-established stemness indices across multiple cancer types: DNA stemness score (DNAss), DNA methylation-based stemness score (DMPss), enhancer-based stemness score (ENHss), and RNA stemness score (RNAss). We analyzed the Spearman correlation between SGO1 expression and DNAss in each tumor. We found a significant correlation in 17 tumors, with a significant positive correlation in 16 tumors like GBM, GBMLGG, LGG, LUAD, BRCA, ESCA, STES, SARC, STAD, PRAD, LIHC, THCA, MESO, PAAD, TGCT, SKCM, and a significant negative correlation in THYM (**Figure S6A**). Subsequently, DMPss analysis revealed that SGO1 expression showed positive correlations in GBM, GBMLGG, LGG, LUAD, LAML, BRCA, ESCA, STES, SARC, STAD, PRAD, THCA, MESO, TGCT, SKCM, and BLCA, while exhibiting a negative correlation in THYM (**Figure S6B**). We also observed that the expression of SGO1 was positively correlated with ENHss in GBM, GBMLGG, LGG, LUAD, BRCA, ESCA, STES, KIPAN, STAD, PRAD, HNSC, LIHC, PAAD, TGCT, SKCM, and negatively correlated in KIRC, THYM, and THCA (**Figure S6C**). Notably, RNAss analysis demonstrated that SGO1 expression exhibited positive correlations in GBM, LGG, CESC, LUAD, COAD, COADREAD, LAML, BRCA, ESCA, STES, SARC, KIRP, STAD, PRAD, UCEC, HNSC, LUSC, THYM, LIHC, READ, PAAD, OV, TGCT, PCPG, SKCM, UVM, BLCA, ACC, KICH, and DLBC, while showing significant negative correlations in KIPAN and THCA (**Figure S6D**). These comprehensive analyses demonstrate that SGO1 expression is strongly associated with tumor stemness across diverse cancer types, suggesting its potential role as a pan-cancer stemness regulator.

### SGO1-related gene enrichment analysis

**Figure 7:**
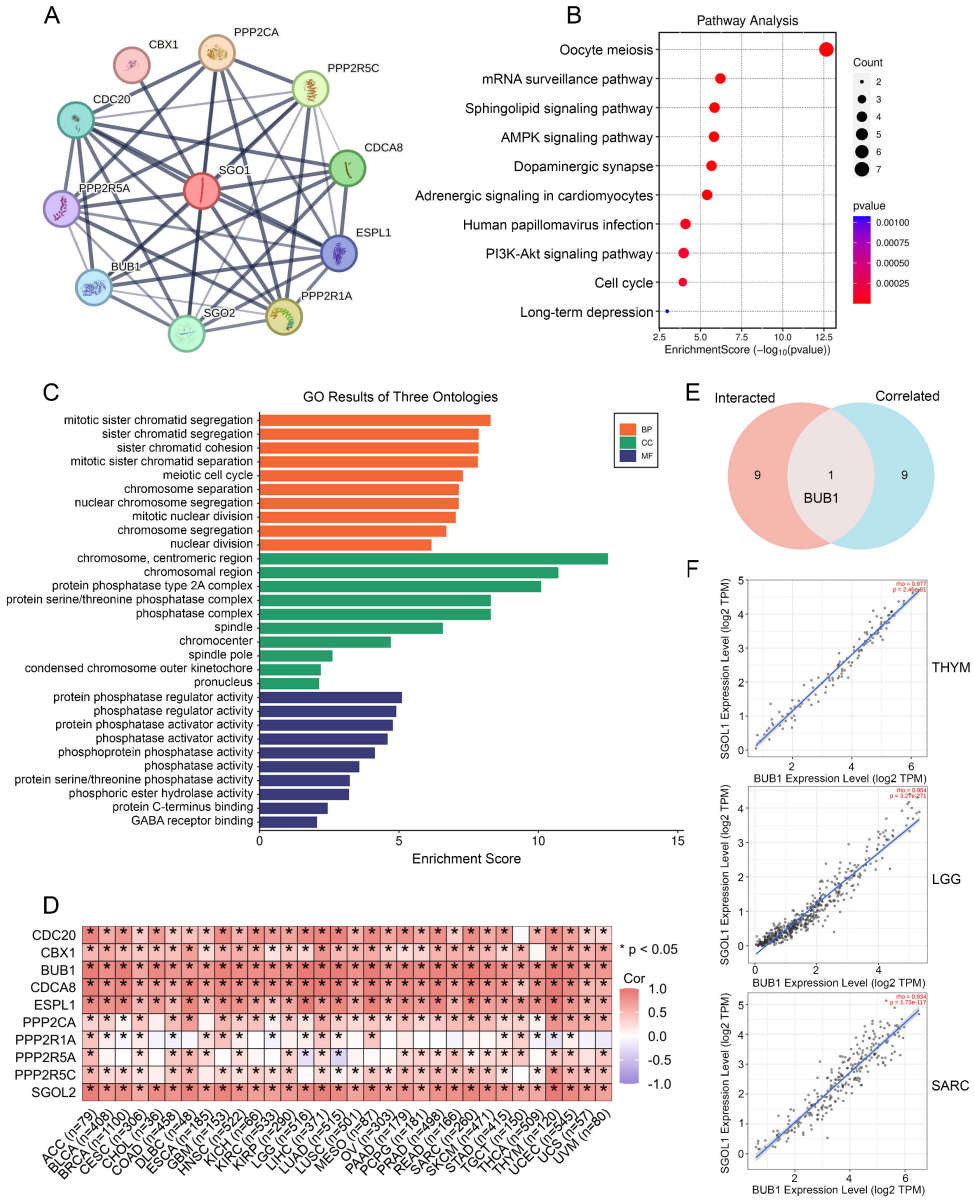
** SGO1 related gene enrichment analysis. (A)** Prediction analysis of SGO1-interacting proteins by using the STRING tool. **(B)** KEGG pathway analysis of SGO1-interacting genes. **(C)** SGO1-related genes were employed to perform GO enrichment analyses. **(D)** Correlation of SGO1 with ten interacting proteins bound by SGO1 in pan-cancer. **(E)** Intersection analysis of SGO1-related genes and SGO1-interaction partners. **(F)** Correlation between SGO1 and BUB1 in the top three tumors of pan-cancer.

SGO1-binding proteins were screened using protein-protein interaction network analysis to further examine the functional mechanism of SGO1 in carcinogenesis. We selected the top ten SGO1-binding proteins provided by the STRING online tool, including those supported or predicted by experimental evidence (**Figure 7A**). Subsequently, we performed KEGG pathway and GO enrichment analysis for the SGO1-interacting top ten genes. According to KEGG pathway analysis, these genes are involved in processes related to oocyte meiosis, mRNA surveillance, and sphingolipid signaling (**Figure 7B**). The biological processes (BP) enrichment results of SGO1-interacting genes revealed that they were mainly associated with sister chromatid segregation/cohesion, meiotic cell cycle, and chromosome segregation (**Figure 7C**). The cellular components (CC) enriched were primarily those related to chromosomal/centromeric region, protein phosphatase type 2A/protein serine/threonine phosphatase complex (**Figure 7C**). Furthermore, the enriched molecular functions (MF) were mainly linked to protein phosphatase regulator activity, phosphatase regulator activity, and protein phosphatase activator activity (**Figure 7C**). Then, GEPIA2 tool was used to summarize all tumor expression data of TCGA, and the top 10 genes related to SGO1 expression were obtained (**Figure 7D**). Excitingly, through intersection analysis, we observed that BUB1 was a common member of these two datasets (**Figure 7E**). Further correlation analysis showed that BUB1 was strongly positively correlated with SGO1 expression in THYM, LGG, and SARC (**Figure 7F**).

### Experimental validation of the function of SGO1 in breast cancer and lung cancer

**Figure 8:**
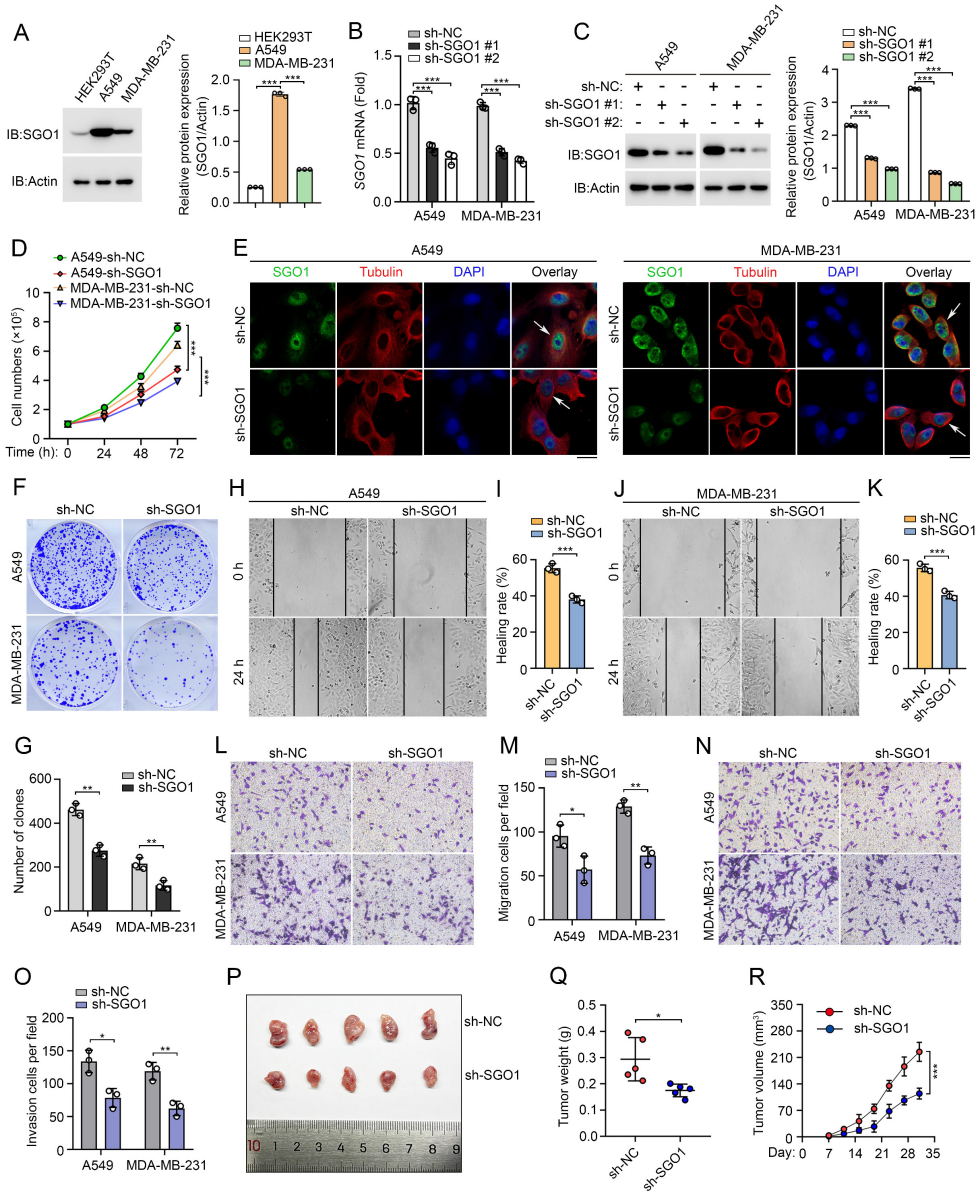
** Experimental validation of the function of SGO1 in breast cancer and lung cancer. (A)** Left: the expression of SGO1 in HEK293T, A549, and MDA-MB-231 cells were detected by Western blot. Right: quantitative grayscale analysis of Western blot bands. **(B)** The knock-down efficiency of SGO1 was detected by RT-PCR. **(C)** Left: Western blot was used to detect the knock-down efficiency of SGO1. Right: quantitative grayscale analysis of Western blot bands. **(D)** Proliferation assay was used to evaluate cell proliferation in MDA-MB-231 and A549 cells. **(E)** Immunofluorescence and DAPI (4',6-Diamidino-2-phenylindole) staining were used to analyze the localization of SGO1 in A549 and MDA-MB-231 cells. Scale bar, 10 μm. **(F, G)** Clone formation assay was performed to evaluate the growth of A549 and MDA-MB-231cells. **(H-M)** Cell scratch and transwell experiments showed that the migration and invasion ability of A549 and MDA-MB-231cells were inhibited after SGO1 knockdown. **(N, O)** Transwell assays were performed to assess cell invasion in A549 and MDA-MB-231cells. **(P-R)** Representative image of subcutaneous tumor representative image, tumor weight, and tumor growth curve of MDA-MB-231 cells xenograft model in SGO1 silenced group and control group, n=5. *P<0.05, **P<0.01, ***P<0.001.

The aforementioned results indicate that SGO1 plays a potentially significant role in promoting tumor growth and migration in breast cancer and lung cancer. To test our hypothesis, we first examined SGO1 protein expression levels in breast cancer cell line MDA-MB-231 and lung cancer cell line A549. We found that SGO1 expression levels were significantly increased in A549 and MDA-MB-231 cells compared to HEK293T cells (**Figure 8A**). Then, knockdown experiments with shRNA showed that SGO1 mRNA transcription levels and protein expression levels were significantly down-regulated in A549 and MDA-MB-231 cells (**Figure 8B, C**). The results of cell proliferation experiments showed that the proliferation of A549 and MDA-MB-231 cells in the SGO1 knockdown group was significantly inhibited compared with the control group (**Figure 8D**). Additionally, knockdown of SGO1 in A549 and MDA-MB-231cells significantly reduced the number of colonies (**Figure 8E, F**). These findings suggested that SGO1 maintains the proliferation and survival of breast and lung cancer cells. Next, the role of SGO1 in cell migration and invasion was examined by cell scratch assay and transwell assay. The results showed that the cell migration of A549 and MDA-MB-231cells with SGO1-silenced was significantly inhibited (**Figure 8G-L**). In the invasion experiment, the cell counts of A549 and MDA-MB-231cells in the SGO1 knockout group were significantly lower than those in the control group (**Figure 8M, N**). Furthermore, the tumor xenograft experiments demonstrated that the tumorigenic potential of the SGO1-silenced group was lower than that of the control mice. The size, weight, and volume of tumors in the treatment group were all significantly lowerer than those in the sh-NC group (**Figure 8O-Q**). Taken together, these data suggest that SGO1 promotes tumor growth and metastasis both *in vitro* and *in vivo*.

## Discussion

SGO1 is a protein that plays a crucial role in cell division and is primarily associated with the regulation and maintenance of the genome during the process of mitosis 31. Emerging research indicates that SGO1 regulates tumorigenesis and progression through various mechanisms, including alterations in expression, signaling pathways, and chromosomal instability 11-14, 32, 33. However, the specific roles of SGO1 in different tumor types and its potential mechanisms remain unclear. To our knowledge, there is currently no pan-cancer analysis of SGO1. Therefore, in this study, we provided a comprehensive bioinformatics analysis of SGO1 in the context of pan-cancer based on multiple databases, examining its expression profiles, prognostic value, genetic mutations, and interaction with the tumor immune microenvironment. Our findings indicate that altered levels of SGO1 expression are significantly associated with cancer development and progression, highlighting its potential role as a pivotal player in tumor biology.

The analysis of gene expression data from TCGA and GTEx indicates that SGO1 was expressed at higher levels in most cancers, including BLCA, BRCA, CESC, COAD, DLBC, ESCA, GBM, LUAD, LUSC, OV, PAAD, READ, SKCM, STAD, THYM, UCEC, and UCS, compared to normal tissues across pan-cancer. In addition, high expression of SGO1 is not only associated with poor prognosis in patients but also closely related to the pathological stages of cancers. These findings suggested that SGO1 may serve as a reliable prognostic biomarker, as its expression levels could inform on disease progression and therapeutic responses. Furthermore, our exploration of gene mutations connected to SGO1 highlights the potential mechanisms through which SGO1 may influence cancer pathophysiology. Mutations affecting SGO1 could disrupt its normal functions in chromosome segregation and DNA repair, contributing to the genomic instability observed in tumors. Understanding these mutations may provide insights into the molecular underpinnings of cancer initiation and progression, revealing targets for therapeutic intervention. A significant aspect of our research focused on the tumor immune microenvironment, specifically through immune cell infiltration, immune checkpoint analysis, and immune scoring. Using the TIMER method, we found that SGO1 is significantly associated with the infiltration levels of various immune cells, including B cells, CD8+ T cells, CD4+ T cells, macrophages, neutrophils, and dendritic cells across different tumor types. Additionally, employing the xCell method, we discovered that the expression of SGO1 is significantly correlated with most of the 38 immune cell subtypes in different tumor types, especially Th2 cells. Immune checkpoint analysis showed that SGO1 was associated with classical immune checkpoints in most tumors, such as PD-1, PD-L1, and CTLA-4. PD-1 and PD-L1 pathways, for instance, are primarily involved in inhibiting T-cell activation, which is a mechanism that many tumors exploit to escape immune surveillance 34, 35. CTLA-4 is another checkpoint that downregulates immune responses, particularly early in T-cell activation 36. For the first time, we reveal positive correlations between SGO1 expression and immune checkpoint genes (PD-1, PD-L1, CTLA4) in most cancers. This finding indicates that SGO1 drives tumor immune escape by upregulating inhibitory checkpoints. This mechanistic link explains poor responses to immunotherapy in SGO1-high tumors and highlights SGO1 as a potential predictor for immunotherapy resistance. Future studies should validate whether SGO1 inhibition synergizes with checkpoint blockade. Such insights are critical, as enhancing anti-tumor immunity could be a viable strategy for improving patient outcomes in cancers characterized by altered SGO1 expression. In addition, the assessment of genomic heterogeneity related to SGO1 provides valuable context for understanding the complexity of cancer. Genomic instability can lead to intratumor heterogeneity, which in turn exhibits strong drug resistance and may lead to poor clinical prognosis 37. Unlike earlier studies focusing on SGO1's mitotic roles, we identified non-canonical associations between SGO1 expression and genomic instability markers (TMB, MSI, HRD). For example, SGO1 positively correlated with HRD in BRCA and LUAD, implying a role in DNA repair dysregulation—a pathway not previously linked to SGO1. By mapping SGO1-correlated genes and their associated protein interaction networks, we have identified that BUB1 was positively correlated with SGO1 expression. BUB1 is a crucial protein involved in the spindle assembly checkpoint (SAC), a critical regulatory mechanism that ensures accurate chromosome segregation during cell division, particularly during mitosis 38, 39. Tumors with co-amplified SGO1/BUB1 exhibited high CIN markers. This subset may be vulnerable to SAC-targeting agents or BUB1 kinase inhibitors currently in preclinical development. Moreover, The SGO1-BUB1 axis may serve as an indirect modulator of immunotherapy response. Existing evidence has established a positive correlation between BUB1 with PD-L1 40, and our pan-cancer analysis confirms SGO1 is linked to PD-L1 across most cancers. These findings suggest dual inhibition of this axis could reverse tumor immune evasion. The SGO1-BUB1 axis represents a novel synthetic lethal node in cancers reliant on mitotic fidelity and immune evasion. Our work provides a rationale for co-targeting these proteins, particularly in aggressive, genomically unstable tumors. Finally, our experimental findings support the relevance of bioinformatics analyses in elucidating the role of SGO1 in cancer progression. The correlation between SGO1 expression and tumor cells behavior evident in both MDA-MB-231 and A549 models, combined with *in vivo* validation through xenografting, underscored SGO1's potential as a key player in tumorigenesis and a promising target for therapeutic intervention. As such, further exploration in both *in vitro* and *in vivo* settings is essential to fully understand the functional consequences of SGO1 regulation and to potentially translate these insights into effective cancer therapies. Notably, previous research has established SGO1 as a crucial mitotic regulator, playing an essential role in safeguarding centromeric cohesion to ensure accurate chromosome segregation 41. This understanding implies that the depletion of SGO1 might be anticipated to impede cell proliferation in both normal and cancer cells, which could be considered a potential limitation of our study. However, our findings have revealed the significance of SGO1 in tumorigenesis and cancer development. SGO1 overexpression in tumors and its altered interaction networks generate cancer-specific vulnerabilities with therapeutic potential. Therefore, we recognize that the relationship between SGO1's physiological function in centromeric cohesion protection and its role as a potential cancer target is complex and requires a dialectical perspective. We believe that our study contributes to a more comprehensive understanding of this intricate relationship, despite the acknowledged potential limitation.

In conclusion, by transcending the traditional view of SGO1 as a mitotic regulator, our work redefines it as a multifunctional orchestrator of tumor progression, immune evasion, and genomic instability. The pan-cancer patterns, immune correlations, and BUB1 interaction uncovered here provide a roadmap for targeting SGO1 in precision oncology, either as a standalone biomarker or in combination with immune/DNA-damaging therapies. By advancing our knowledge of SGO1, we can potentially unlock new strategies for cancer diagnosis and treatment, ultimately improving outcomes for patients with malignancies exhibiting altered SGO1 expression.

## Supplementary Material

Supplementary figures.

## References

[1] Bray F, Ferlay J, Soerjomataram I, Siegel RL, Torre LA, Jemal A (2018). Global cancer statistics 2018: GLOBOCAN estimates of incidence and mortality worldwide for 36 cancers in 185 countries. CA Cancer J Clin.

[2] Santucci C, Carioli G, Bertuccio P, Malvezzi M, Pastorino U, Boffetta P (2020). Progress in cancer mortality, incidence, and survival: a global overview. Eur J Cancer Prev.

[3] Li W, Liu J (2022). The Prognostic and Immunotherapeutic Significance of AHSA1 in Pan-Cancer, and Its Relationship with the Proliferation and Metastasis of Hepatocellular Carcinoma. Front Immunol.

[4] Saidak Z, Soudet S, Lottin M, Salle V, Sevestre MA, Clatot F (2021). A pan-cancer analysis of the human tumor coagulome and its link to the tumor immune microenvironment. Cancer Immunol Immunother.

[5] Wang Z, Zhang H, Cheng Q (2020). PDIA4: The basic characteristics, functions and its potential connection with cancer. Biomed Pharmacother.

[6] Yamada HY, Yao Y, Wang X, Zhang Y, Huang Y, Dai W (2012). Haploinsufficiency of SGO1 results in deregulated centrosome dynamics, enhanced chromosomal instability and colon tumorigenesis. Cell Cycle.

[7] Zhang Q, Liu H (2020). Functioning mechanisms of Shugoshin-1 in centromeric cohesion during mitosis. Essays Biochem.

[8] Iwaizumi M, Shinmura K, Mori H, Yamada H, Suzuki M, Kitayama Y (2009). Human Sgo1 downregulation leads to chromosomal instability in colorectal cancer. Gut.

[9] Yang L, Zhang Q, Niu T, Liu H (2021). SET levels contribute to cohesion fatigue. Mol Biol Cell.

[10] Mu J, Fan L, Liu D, Zhu D (2019). Overexpression of shugoshin1 predicts a poor prognosis for prostate cancer and promotes metastasis by affecting epithelial-mesenchymal transition. Onco Targets Ther.

[11] Chen Q, Wan X, Chen Y, Liu C, Gu M, Wang Z (2019). SGO1 induces proliferation and metastasis of prostate cancer through AKT-mediated signaling pathway. Am J Cancer Res.

[12] Matsuura S, Kahyo T, Shinmura K, Iwaizumi M, Yamada H, Funai K (2013). SGOL1 variant B induces abnormal mitosis and resistance to taxane in non-small cell lung cancers. Sci Rep.

[13] Yuan Y, Wang J, Zhang D, Tang L, Duan L, Jiang X (2022). Deciphering the Role of Shugoshin-Like Protein 1 in Lung Adenocarcinoma: A Comprehensive Analysis and *In Vitro* Study. Front Oncol.

[14] Jusino S, Rivera-Rivera Y, Chardon-Colon C, Rodriguez-Rodriguez PC, Roman-Gonzalez J, Julia-Hernandez VS (2023). Sustained Shugoshin 1 downregulation reduces tumor growth and metastasis in a mouse xenograft tumor model of triple-negative breast cancer. Cell Div.

[15] Sun W, He B, Yang B, Hu W, Cheng S, Xiao H (2018). Genome-wide CRISPR screen reveals SGOL1 as a druggable target of sorafenib-treated hepatocellular carcinoma. Lab Invest.

[16] Li T, Fu J, Zeng Z, Cohen D, Li J, Chen Q (2020). TIMER2.0 for analysis of tumor-infiltrating immune cells. Nucleic Acids Res.

[17] Tang Z, Li C, Kang B, Gao G, Li C, Zhang Z (2017). GEPIA: a web server for cancer and normal gene expression profiling and interactive analyses. Nucleic Acids Res.

[18] Uhlen M, Fagerberg L, Hallstrom BM, Lindskog C, Oksvold P, Mardinoglu A (2015). Proteomics. Tissue-based map of the human proteome. Science.

[19] Chandrashekar DS, Bashel B, Balasubramanya SAH, Creighton CJ, Ponce-Rodriguez I, Chakravarthi B (2017). UALCAN: A Portal for Facilitating Tumor Subgroup Gene Expression and Survival Analyses. Neoplasia.

[20] Gao J, Aksoy BA, Dogrusoz U, Dresdner G, Gross B, Sumer SO (2013). Integrative analysis of complex cancer genomics and clinical profiles using the cBioPortal. Sci Signal.

[21] Liu CJ, Hu FF, Xia MX, Han L, Zhang Q, Guo AY (2018). GSCALite: a web server for gene set cancer analysis. Bioinformatics.

[22] Shen W, Song Z, Zhong X, Huang M, Shen D, Gao P (2022). Sangerbox: A comprehensive, interaction-friendly clinical bioinformatics analysis platform. Imeta.

[23] Szklarczyk D, Kirsch R, Koutrouli M, Nastou K, Mehryary F, Hachilif R (2023). The STRING database in 2023: protein-protein association networks and functional enrichment analyses for any sequenced genome of interest. Nucleic Acids Res.

[24] Tang D, Chen M, Huang X, Zhang G, Zeng L, Zhang G (2023). SRplot: A free online platform for data visualization and graphing. PLoS One.

[25] Hanahan D, Weinberg RA (2011). Hallmarks of cancer: the next generation. Cell.

[26] Ward PS, Thompson CB (2012). Metabolic reprogramming: a cancer hallmark even warburg did not anticipate. Cancer Cell.

[27] Fridman WH, Pages F, Sautes-Fridman C, Galon J (2012). The immune contexture in human tumours: impact on clinical outcome. Nat Rev Cancer.

[28] Gong T, Borgard H, Zhang Z, Chen S, Gao Z, Deng Y (2022). Analysis and Performance Assessment of the Whole Genome Bisulfite Sequencing Data Workflow: Currently Available Tools and a Practical Guide to Advance DNA Methylation Studies. Small Methods.

[29] Huang W, Li H, Yu Q, Xiao W, Wang DO (2022). LncRNA-mediated DNA methylation: an emerging mechanism in cancer and beyond. J Exp Clin Cancer Res.

[30] Mattei AL, Bailly N, Meissner A (2022). DNA methylation: a historical perspective. Trends Genet.

[31] Watanabe Y (2005). Shugoshin: guardian spirit at the centromere. Curr Opin Cell Biol.

[32] Rao CV, Sanghera S, Zhang Y, Biddick L, Reddy A, Lightfoot S (2016). Systemic Chromosome Instability Resulted in Colonic Transcriptomic Changes in Metabolic, Proliferation, and Stem Cell Regulators in Sgo1-/+ Mice. Cancer Res.

[33] Rao CV, Sanghera S, Zhang Y, Biddick L, Reddy A, Lightfoot S (2016). Antagonizing pathways leading to differential dynamics in colon carcinogenesis in Shugoshin1 (Sgo1)-haploinsufficient chromosome instability model. Mol Carcinog.

[34] Doroshow DB, Bhalla S, Beasley MB, Sholl LM, Kerr KM, Gnjatic S (2021). PD-L1 as a biomarker of response to immune-checkpoint inhibitors. Nat Rev Clin Oncol.

[35] Lin X, Kang K, Chen P, Zeng Z, Li G, Xiong W (2024). Regulatory mechanisms of PD-1/PD-L1 in cancers. Mol Cancer.

[36] Chikuma S (2017). CTLA-4, an Essential Immune-Checkpoint for T-Cell Activation. Curr Top Microbiol Immunol.

[37] Dagogo-Jack I, Shaw AT (2018). Tumour heterogeneity and resistance to cancer therapies. Nat Rev Clin Oncol.

[38] Carvalhal S, Bader I, Rooimans MA, Oostra AB, Balk JA, Feichtinger RG (2022). Biallelic BUB1 mutations cause microcephaly, developmental delay, and variable effects on cohesion and chromosome segregation. Sci Adv.

[39] Vanoosthuyse V, Hardwick KG (2005). Bub1 and the multilayered inhibition of Cdc20-APC/C in mitosis. Trends Cell Biol.

[40] Zhang L, Zhuge Y, Ni J (2025). BUB1 serves as a biomarker for poor prognosis in liver hepatocellular carcinoma. BMC Immunol.

[41] Watanabe Y, Kitajima TS (2005). Shugoshin protects cohesin complexes at centromeres. Philos Trans R Soc Lond B Biol Sci.

