# Supplementary material for: Pan-cancer analysis reveals SGO1 as a potential cancer prognostic and immunological biomarker
Source: J Cancer. 2025 Jul 24;16(11):3372–88. doi: 10.7150/jca.115334 (PMC12374945; doi:10.7150/jca.115334)
Supplement: Supplementary file 1 — Supplementary figures. [file jcav16p3372s1.pdf]

Figure S1. Expression and subcellular localization of SGO1 in cancers.

(A) Protein expression of SGO1 in breast, lung, stomach, liver, colorectum, and ovary cancers and normal tissues from the HPA database. (B) Immunofluorescence staining of the subcellular localization of SGO1 was analyzed in U-251MG, U2OS, and PC-3 cells from the HPA database.

Figure S2. Prognostic significance of SGO1 in different types of human cancers using the Kaplan-Meier plotter database.

(A-I) Kaplan-Meier plots showed that the SGO1 expression levels were associated with overall survival (OS) in BRCA, EAC, KIRC, KIRP, LIHC, LUAD, PADC, SARC, and UCEC. (J-R) Kaplan-Meier plots showed that the SGO1 expression levels were associated with recurrence free survival (RFS) in CESC, EAC, KIRP, LIHC, LUAD, PADC, SARC, THCA, and UCEC.

Figure S3. DNA methylation level of SGO1 in pan-cancer.

(A-K) Promoter methylation level of SGO1 in BLCA, LIHC, LUAD, LUSC, PRAD, TGCT, THCA, UCEC, CHOL, KIRC and SARC. *$p < 0.05$, **$p < 0.01$, ***$p < 0.001$.

Figure S4. Mutation feature of SGO1 in pan-cancer.

(A) Mutation types and alteration frequencies of SGO1 in pan-cancer. (B) Genetic alterations of SGO1, including types, locations, and frequencies.

Figure S5. Genomic heterogeneity analysis of SGO1.

(A-G) Correlation analysis of SGO1 expression with TMB, MATH, MSI, purity, HDR, LOH, and ploidy in pan-cancer

Figure S6. Analysis of SGO1 in tumor stemness

(A-D) Correlation analysis of SGO1 expression with tumor stemness indicators DNAss, DMPss, ENHss, and RNAss in pan-cancer.

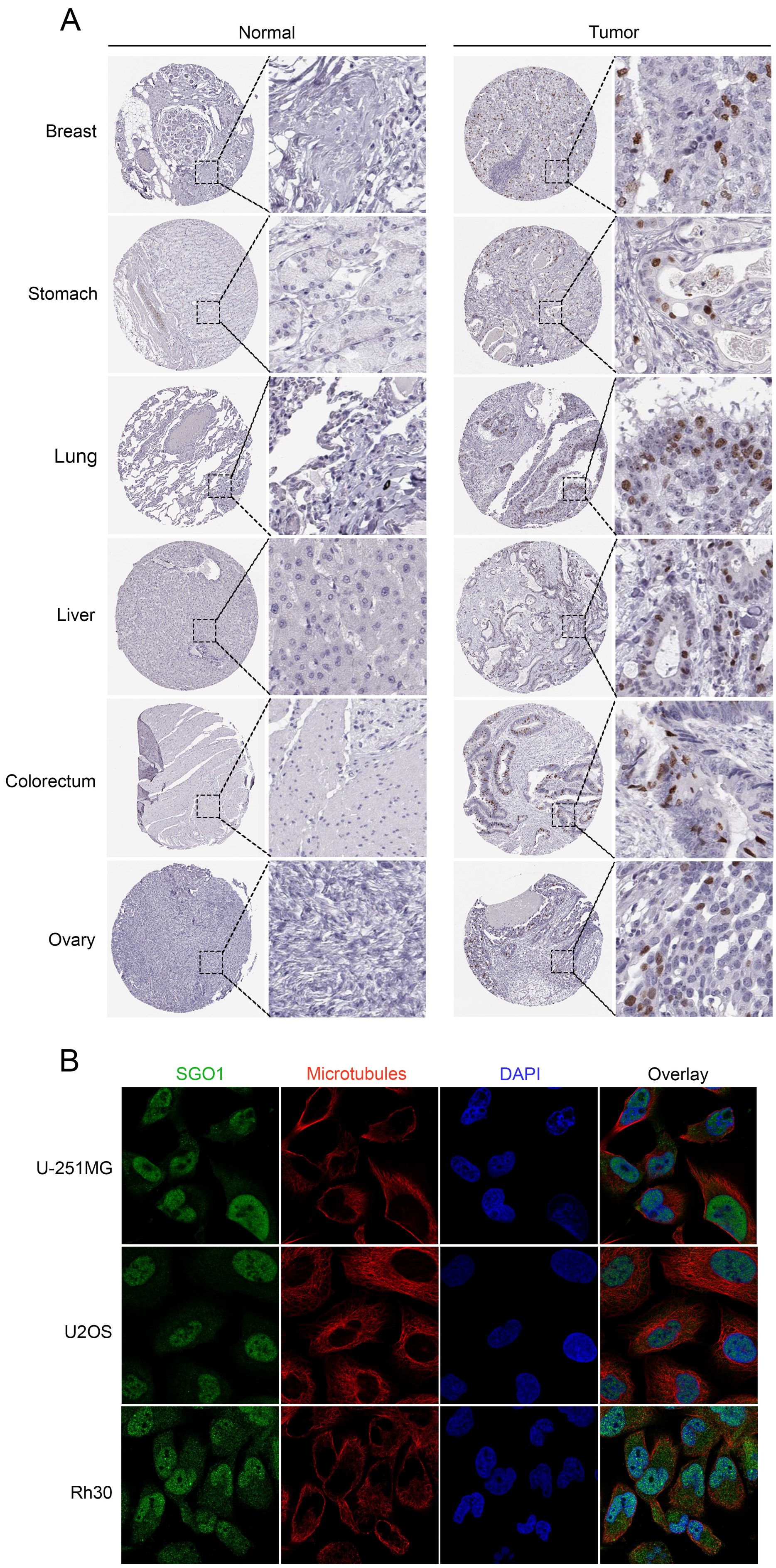

Supplementary figure 1

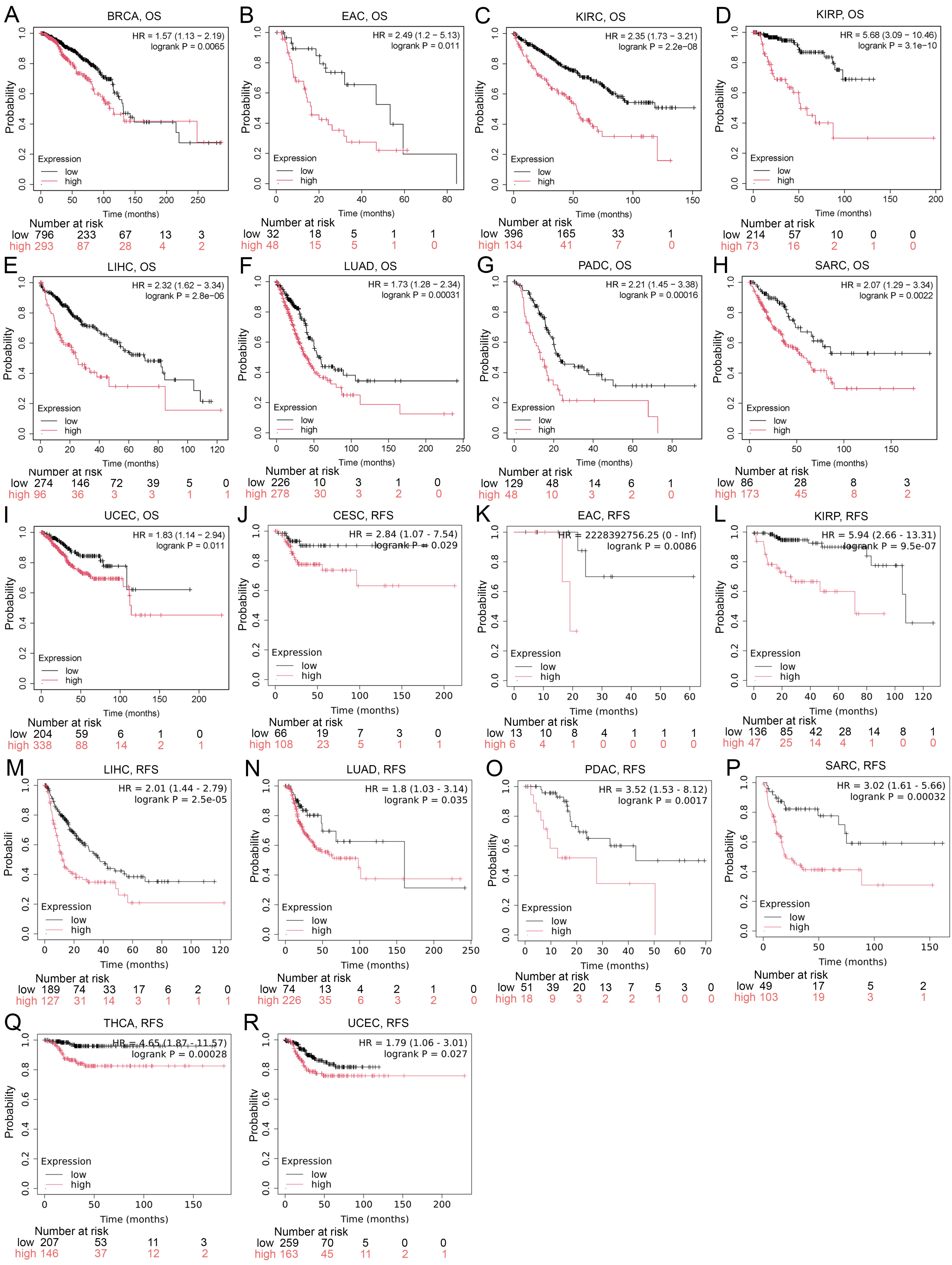

Supplementary figure 2

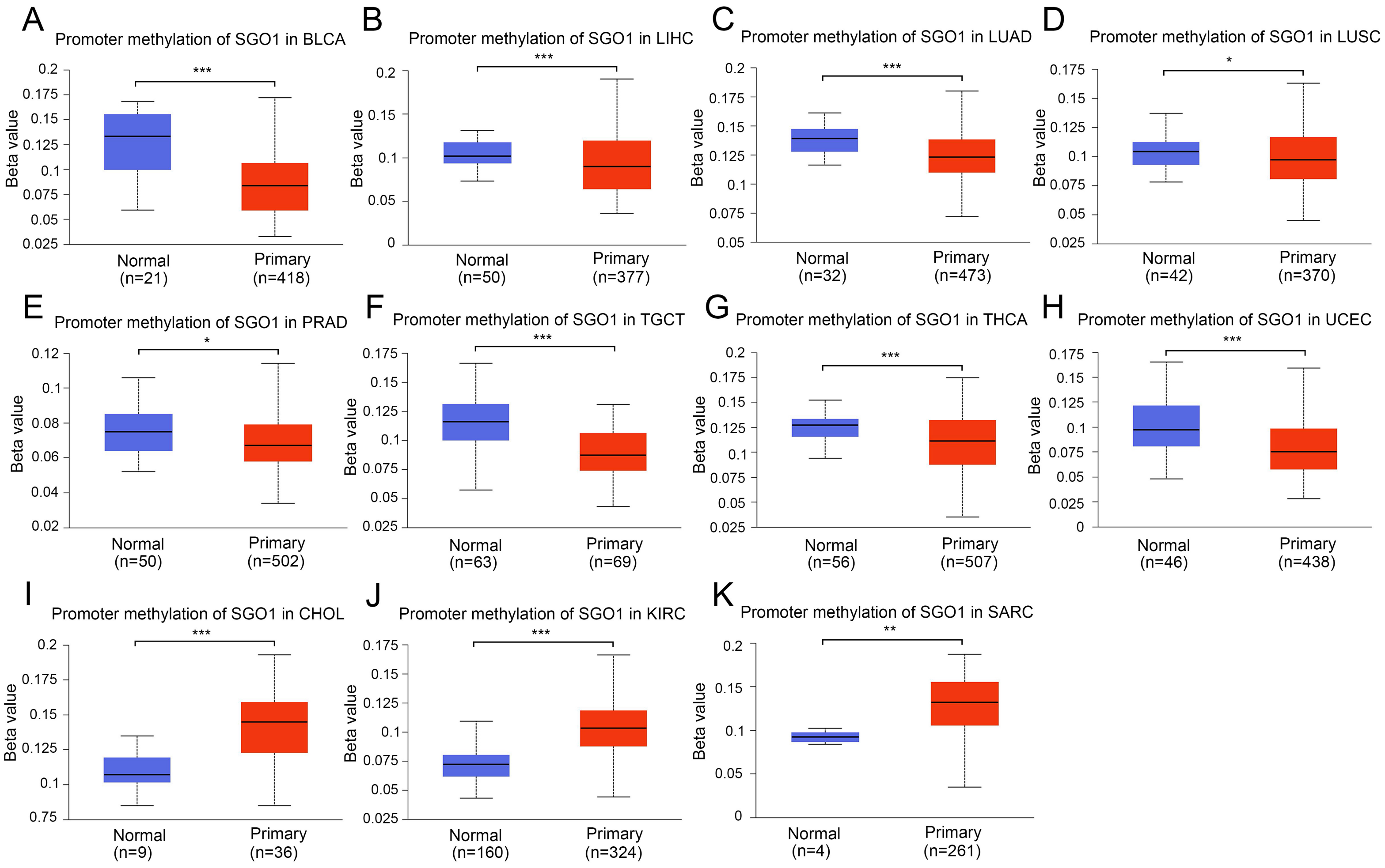

Supplementary figure 3

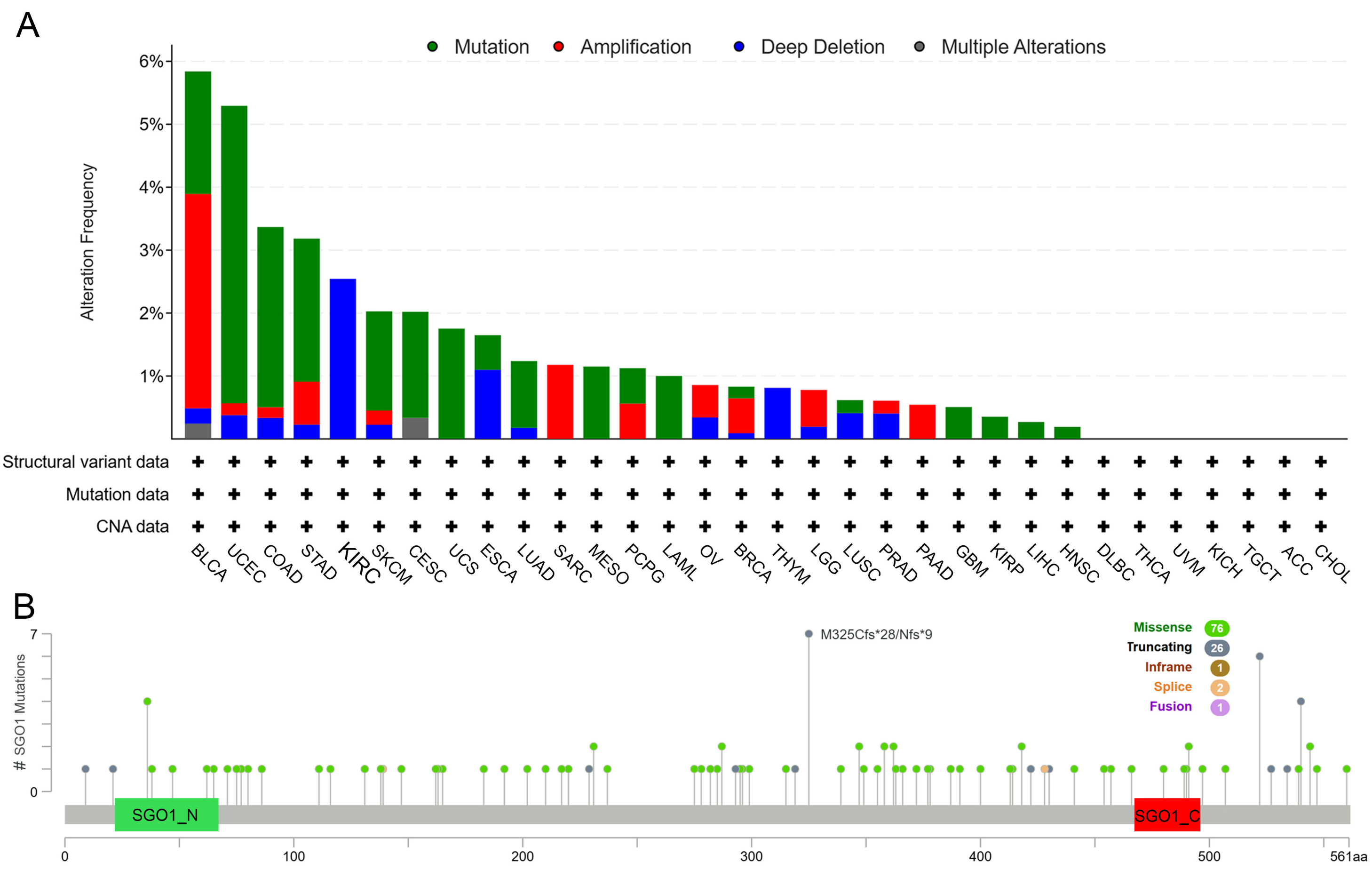

Supplementary figure 4

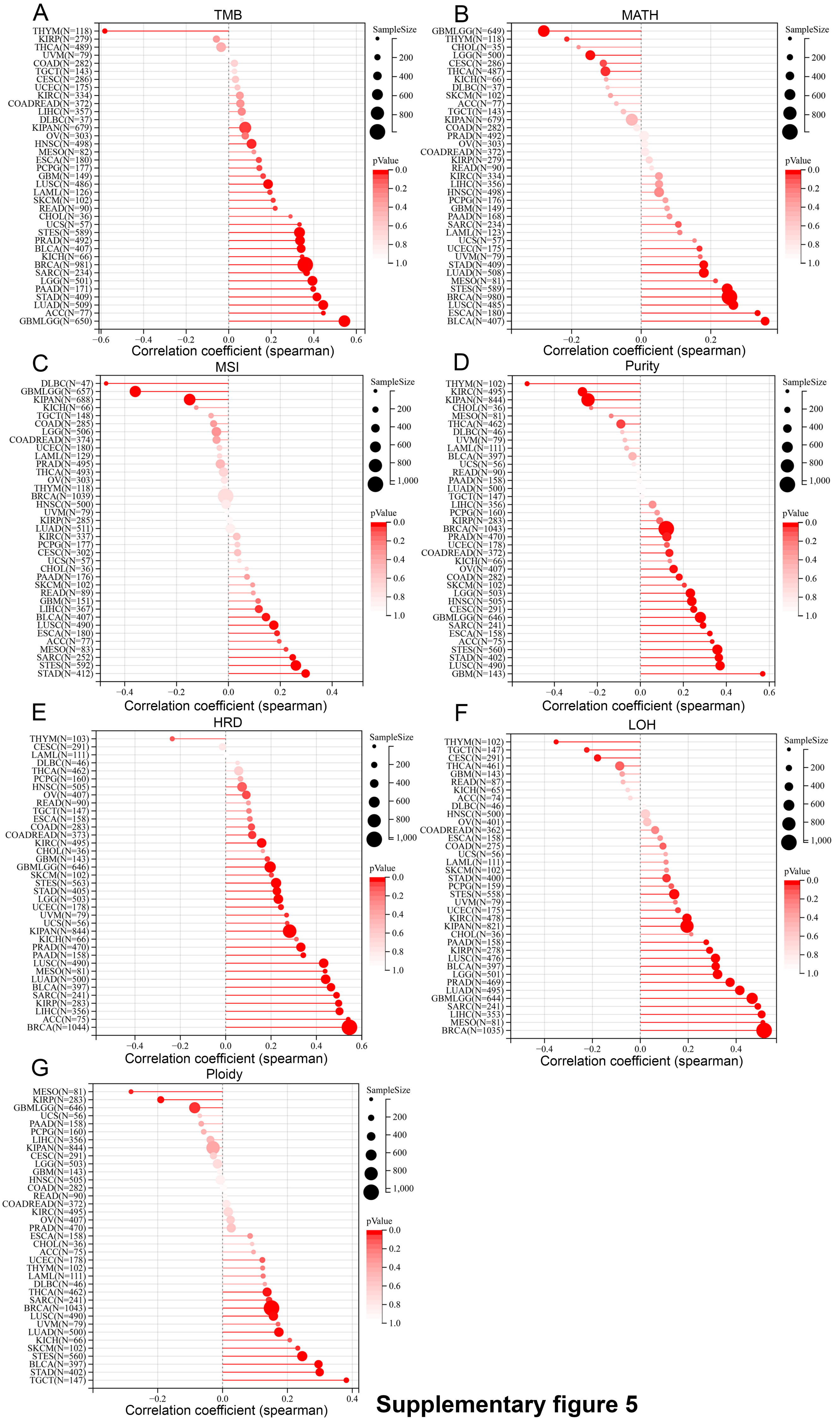

Supplementary figure 5

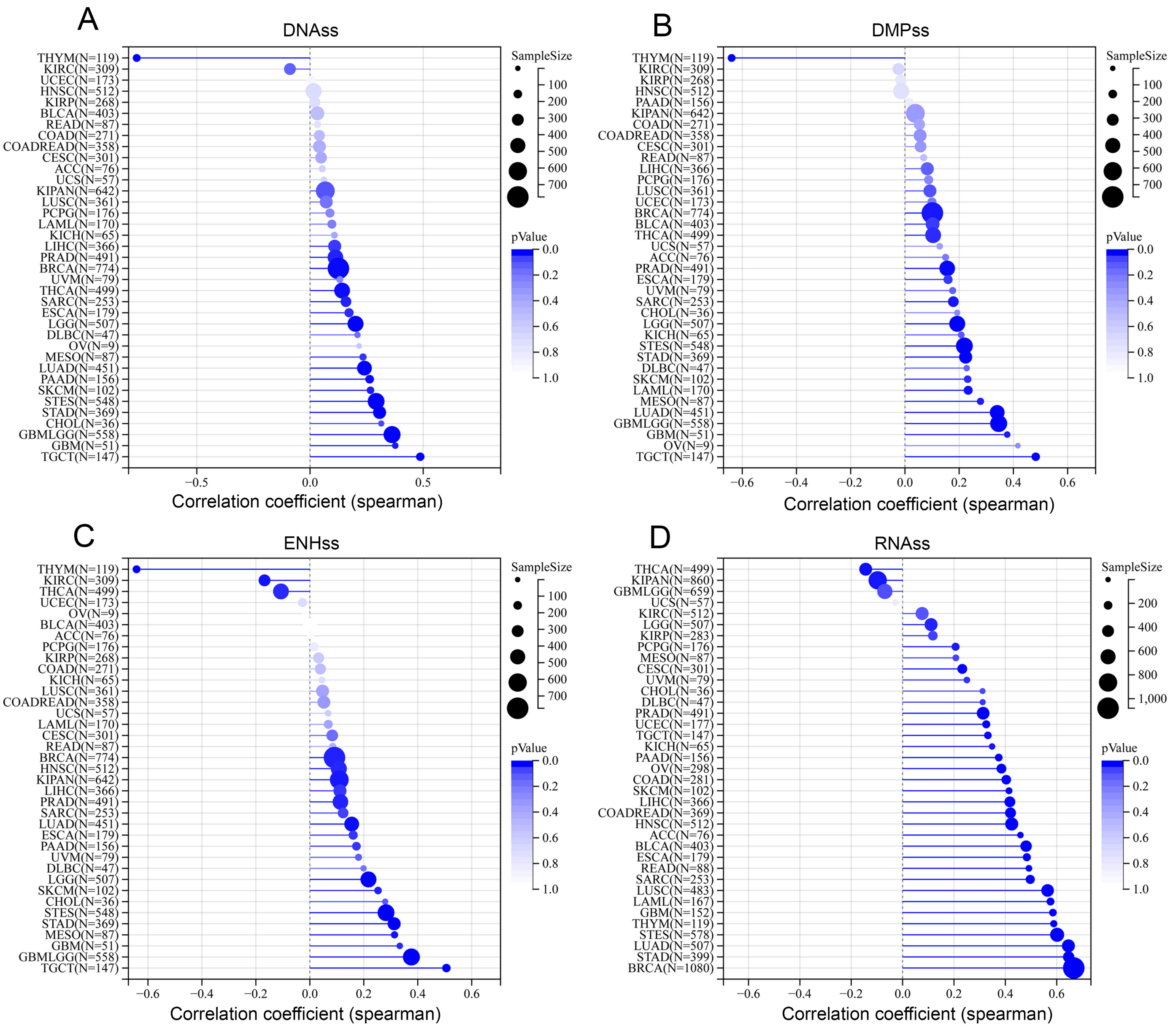

Supplementary figure 6
